# Four missense genetic variants in *CUBN* are associated with higher levels of eGFR in non-diabetes but not in diabetes mellitus or its subtypes: A genetic association study in Europeans

**DOI:** 10.3389/fendo.2023.1081741

**Published:** 2023-02-28

**Authors:** Nicoline Uglebjerg, Fariba Ahmadizar, Dina M. Aly, Marisa Cañadas-Garre, Claire Hill, Annemieke Naber, Asmundur Oddsson, Sunny S. Singh, Laura Smyth, David-Alexandre Trégouët, Layal Chaker, Mohsen Ghanbari, Valgerdur Steinthorsdottir, Emma Ahlqvist, Samy Hadjadj, Mandy Van Hoek, Maryam Kavousi, Amy Jayne McKnight, Eric J. Sijbrands, Kari Stefansson, Matias Simons, Peter Rossing, Tarunveer S. Ahluwalia

**Affiliations:** ^1^ Complications Research, Steno Diabetes Center Copenhagen, Herlev, Denmark; ^2^ Department of Epidemiology, Erasmus Medical Center, University Medical Center Rotterdam, Rotterdam, Netherlands; ^3^ Department of Data Science & Biostatistics, Julius Global Health, University Medical Center Utrecht, Utrecht, Netherlands; ^4^ Department of Clinical Sciences, Lund University, Malmö, Sweden; ^5^ Centre for Public Health, Queen’s University Belfast, Belfast, United Kingdom; ^6^ GENYO Centre for Genomics and Oncological Research, Pfizer-University of Granada-Andalusian Regional Government, Granada, Spain; ^7^ Instituto de Investigación Biosanitaria de Granada (ibs.GRANADA), Granada, Spain; ^8^ Department of Internal Medicine, Erasmus Medical Center, University Medical Center Rotterdam, Rotterdam, Netherlands; ^9^ deCODE Genetics, Amgen, Inc., Reykjavik, Iceland; ^10^ University of Bordeaux, Institut National de la Santé et de la Recherche Médicale (INSERM), Bordeaux Population Health Research Center, Bordeaux, France; ^11^ Nantes Université, Centre Hospitalier Universitaire Nantes, Centre National de la Recherche Scientifique, INSERM, l’institut du thorax, Nantes, France; ^12^ Faculty of Medicine, School of Health Sciences, University of Iceland, Reykjavik, Iceland; ^13^ Institute of Human Genetics, University Hospital Heidelberg, Heidelberg, Germany; ^14^ Department of Clinical Medicine, University of Copenhagen, Copenhagen, Denmark; ^15^ The Bioinformatics Center, Department of Biology, University of Copenhagen, Copenhagen, Denmark

**Keywords:** genetics, *CUBN*, cubilin, kidney function, eGFR, diabetes, non-diabetes, chronic kidney disease (CKD)

## Abstract

**Aim:**

Rare genetic variants in the *CUBN* gene encoding the main albumin-transporter in the proximal tubule of the kidneys have previously been associated with microalbuminuria and higher urine albumin levels, also in diabetes. Sequencing studies in isolated proteinuria suggest that these variants might not affect kidney function, despite proteinuria. However, the relation of these *CUBN* missense variants to the estimated glomerular filtration rate (eGFR) is largely unexplored. We hereby broadly examine the associations between four *CUBN* missense variants and eGFR_creatinine_ in Europeans with Type 1 (T1D) and Type 2 Diabetes (T2D). Furthermore, we sought to deepen our understanding of these variants in a range of single- and aggregate- variant analyses of other kidney-related traits in individuals with and without diabetes mellitus.

**Methods:**

We carried out a genetic association-based linear regression analysis between four *CUBN* missense variants (*rs141640975*, *rs144360241*, *rs45551835*, *rs1801239*) and eGFR_creatinine_ (ml/min/1.73 m^2^, CKD-EPI_creatinine(2012)_, natural log-transformed) in populations with T1D (n ~ 3,588) or T2D (n ~ 31,155) from multiple European studies and in individuals without diabetes from UK Biobank (UKBB, n ~ 370,061) with replication in deCODE (n = 127,090). Summary results of the diabetes-group were meta-analyzed using the fixed-effect inverse-variance method.

**Results:**

Albeit we did not observe associations between eGFR_creatinine_ and *CUBN* in the diabetes-group, we found significant positive associations between the minor alleles of all four variants and eGFR_creatinine_ in the UKBB individuals without diabetes with *rs141640975* being the strongest (Effect=0.02, P_eGFR_creatinine_=2.2 × 10^-9^). We replicated the findings for *rs141640975* in the Icelandic non-diabetes population (Effect=0.026, P_eGFR_creatinine_=7.7 × 10^-4^). For *rs141640975*, the eGFR_creatinine_-association showed significant interaction with albuminuria levels (normo-, micro-, and macroalbuminuria; p = 0.03). An aggregated genetic risk score (GRS) was associated with higher urine albumin levels and eGFR_creatinine_. The *rs141640975* variant was also associated with higher levels of eGFR_creatinine-cystatin C_ (ml/min/1.73 m^2^, CKD-EPI_2021_, natural log-transformed) and lower circulating cystatin C levels.

**Conclusions:**

The positive associations between the four *CUBN* missense variants and eGFR in a large population without diabetes suggests a pleiotropic role of *CUBN* as a novel eGFR-locus in addition to it being a known albuminuria-locus. Additional associations with diverse renal function measures (lower cystatin C and higher eGFR_creatinine-cystatin C_ levels) and a *CUBN*-focused GRS further suggests an important role of *CUBN* in the future personalization of chronic kidney disease management in people without diabetes.

## 1 Introduction

Urine albumin or albuminuria is one of the most important biomarkers of kidney damage in individuals with or without diabetes. In healthy individuals, the glomerular filter in the kidneys retains most of the albumin, although a small amount can usually pass through to the tubular system (1). Reabsorption of albumin is facilitated by the kidney’s proximal tubular cells (PTCs), ensuring that almost no albumin is excreted in urine under normal conditions (2, 3). Elevated excretion of albumin in the urine - initially coined as “microalbuminuria” - is one of the earliest signs of chronic kidney disease (CKD) and may be the kidney-related manifestation of general endothelial damage, where scarring of the glomerulus causes chronic leakiness through the filter of albumin and other proteins (4).

Over the past decades, the number of people with diabetes mellitus has more than doubled to a global prevalence of 537 million in 2021 (5), with serious consequences for the healthcare system and society. According to a recent European study (6), one in four hospitalized patients has diabetes. Up to 40% of individuals with diabetes develop diabetic kidney disease (DKD), which is associated with elevated cardiovascular morbidity and mortality and progresses to dependency on kidney replacement therapies such as dialysis and transplantation and is a leading cause of CKD (7).

In the recent years, studies have begun to unravel genetic aspects of albuminuria. Recently, we and others identified that genetic variants (single nucleotide variants (SNVs)) in the gene encoding for cubilin (*CUBN*) – the main albumin-transporter in PTCs (1, 8) – are associated with microalbuminuria and higher urine albumin levels in populations with and without diabetes (8–14). Four variants in the C-terminal end of cubilin have been of particular interest (*rs141640975 (c.5069C>T; p.Ala1690Val)*, *rs144360241 (c.6469A>G; p.Asn2157Asp)*, *rs45551835 (c.8741C>T; p.Ala2914Val)*, and *rs1801239 (c.8950A>G, p.Ile2984Val)*); these are functional (missense) variants that have been proposed to alter the function of cubilin, leading to a form of albuminuria that may reflect a lack of tubular reabsorption of albumin (i.e., tubular albuminuria) (8). *In silico* structural and damage prediction analyses of the variants indicate their potential to change secondary or even tertiary structure(s) in the cubilin protein and to have different degrees of damaging effects on protein function, disease, or both (8). Our recent study further suggests that the effect of some of these variants on urine albumin levels is 2-3 times higher in diabetes compared to non-diabetes (11).

However, the role of these *CUBN* variants in relation to estimated glomerular filtration rate (eGFR), a clinically used marker of kidney function, is largely unexplored, and most genetic studies have focused on the general population (8, 9, 11). Recent efforts to uncover the role of these variants specifically in diabetes – and to clearly separate the effect seen here from the effect in the non-diabetes-proportion of the general population – have been performed as relatively small secondary analyses without including *rs144360241* or diabetes subtypes (8). Thus far, only *rs45551835* has been connected to higher levels of eGFR in type 2 diabetes and *rs141640975* in non-diabetes (8). Therefore, we investigated the relationship between the four *CUBN* variants and eGFR in different contexts: First, we meta-analyzed studies of SNV-eGFR_creatinine_ regressions in Europeans with type 1 (T1D) or type 2 diabetes mellitus (T2D). We then examined single- and aggregate-variant associations separately in diabetes and non-diabetes populations of a large, nationally representative cohort facilitating application of identical phenotype definitions, including the dependency of albuminuria-stage in SNV-eGFR_creatinine_ associations, generation of a *CUBN*-specific genetic risk score (GRS), and identification of associations between individual SNVs and cystatin C-based measures of kidney function. Together, these analyses both seek to replicate previous associations in DM and NDM populations and to provide novel insights into the link between *CUBN* and eGFR.

## 2 Methods

### 2.1 Study design and cohorts

**Figure 1:**
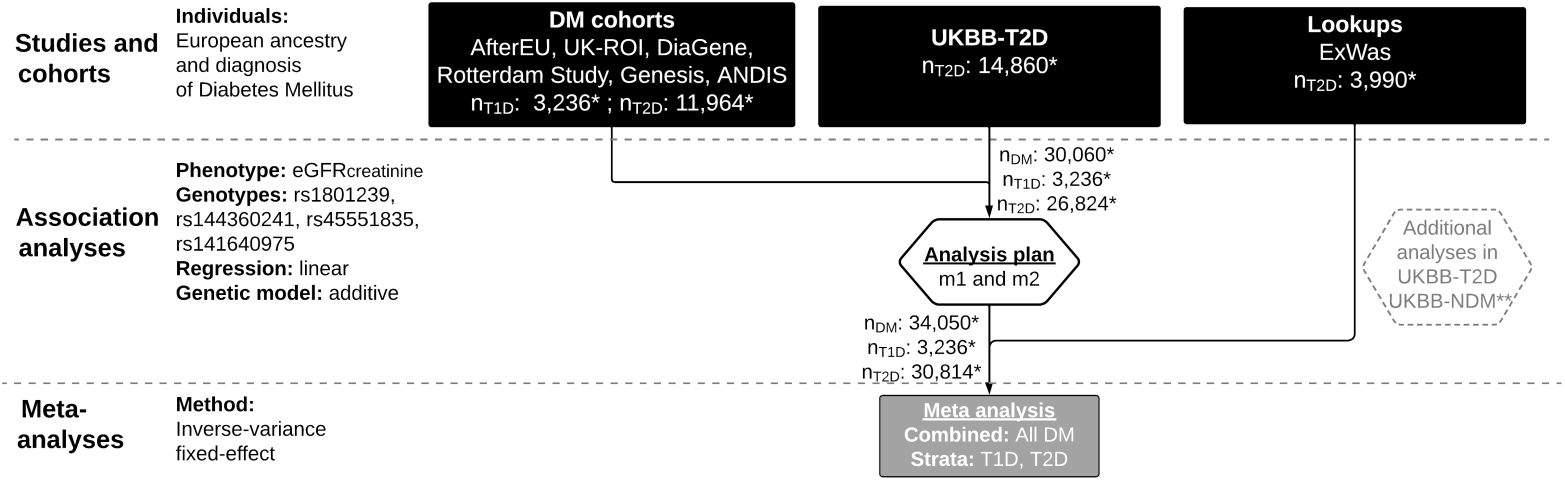
Flow chart of SNV-eGFRcreatinine meta-analyses in Diabetes. UKBB, UK Biobank; T2D, Type 2 diabetes; DM, diabetes mellitus; T1D, Type 1 diabetes; NDM, without diabetes mellitus; SNV, single nucleotide variant; eGFRcreatinine, Estimated glomerular filtration rate, natural log-transformed; PCs, Principal components of population structure; HbA1C, hemoglobin A1C; SBP, Systolic blood pressure; m1: model 1 (eGFRcreatinine ~ genotype + sex + age + 0-10 PCs); m2: model 2 (m1 + HbA1c + SBP + diabetes duration); * Sample sizes (n) reflect the maximal number of individuals (out of the total number of individuals in **Table 1**) available for rs45551835, model 1. ** See **Supplementary Figure 1** for a flow chart of additional analyses. Figure made with LucidChart (lucid.app).

For the genetic association meta-analysis in diabetes mellitus (DM), we included data collected *via* three approaches (**Figure 1**): First, we acquired summary statistics from up to 15,200 individuals of European origin with either type 1 diabetes (T1D) or type 2 diabetes (T2D) subsetted from six cohorts: AfterEU (T1D) (15–18), Rotterdam (T2D) (19), DiaGene (T2D) (20), UK-ROI (T1D) (21), Genesis (T1D) (22) and ANDIS (T2D) (23). These studies (hereafter referred to as “DM cohorts”) were invited to the study and given a harmonized analysis plan provided that any subset of the requested genetic variants was available. A description of each cohort can be found in the **Supplemental text**.

Second, we applied the same analysis plan to a subset of individuals with T2D (n ~ 14,860) from the UK Biobank (24) (henceforth referred to as “UKBB-T2D”). The approach we used to extract the T2D subset has been described previously (25, 26).

Third, we did a lookup in a subset of an exome-wide association study (henceforth referred to as “ExWas”) that included 3,990 individuals with T2D from three Danish studies (Inter99, Vejle biobank and Addition-DK) described previously (11).

We also searched the Type 2 Diabetes Knowledge Portal [at time of search: www.type2diabetesgenetics.org, now: https://t2d.hugeamp.org/ (27)] for large-scale studies with publicly available summary statistics fulfilling the following criteria: Summary statistics should *a)* be readily available through the knowledge portal or a direct link to a study website; *b)* be available for diabetes-stratified and European-only populations; *c)* include at least one target genotype; *d)* be based on natural log-transformed eGFR values rather than non-transformed eGFR values; and *e)* be based on regression models with covariate adjustments comparable to those in the other cohorts in this study. However, as of 10 July 2020, no studies in the portal fulfilled our criteria, and no additional studies were included.

For additional analyses, we used *1)* a group of individuals without diabetes from UKBB (n ~ up to 370,000 individuals), henceforth referred to as “UKBB-NDM”) and *2)* the UKBB-T2D group, which was also part of the meta-analysis (**Supplementary Figure 1**). 127,090 non-diabetes individuals from the Icelandic study deCODE participated as the replication cohort (**Supplemental text**).

This research work was conducted in accordance with the Helsinki Declaration. Ethical approval was previously obtained locally for individual studies. All participants gave written informed consent before participating.

### 2.2 Phenotype details

For the DM cohorts and UKBB (both NDM and T2D groups), we calculated the creatinine-based estimated glomerular filtration rate (eGFR_creatinine_) with the Chronic Kidney Disease Epidemiology Collaboration creatinine equation (CKD-EPI_creatinine(2012)_, ml/min/1.73 m^2^ (28), natural log-transformed). We included it here as a continuous variable. Other measures of kidney function were also calculated for UKBB; see section 2.4.2.4.

### 2.3 Genotyping, imputation, quality control and variant selection

We obtained information on genotyping, imputation, and quality control of each cohort and summarized it in **Supplementary Tables 1**, **2**.

Four variants were selected for further analysis: *rs141640975* (Chromosome (chr) 10, position (pos) 16992011 (genome-build GRCh37.p13)) with minor allele frequency (MAF) 0.002-0.009; *rs144360241* (chr 10, pos 16967417) with MAF 0.006-0.010; *rs45551835* (chr 10, pos 16932384) with MAF 0.016-0.021; and *rs1801239* (chr 10, pos 16919052) with MAF 0.097-0.114. For the deCODE study, the MAFs were in the same range except *rs144360241* (MAF: 0.002). The minor alleles of these variants (A, C, A, and C, respectively) were used as effect alleles.

We used LDlink version 5.1 (29) with the European (CEU + GBR) reference panel to confirm the independent relationship (Linkage Disequilibrium (LD) r^2^< 0.1) between these SNVs.

The SNVs were first used in single-variant analyses and were then combined into a genetic risk score (GRS; see description below).

### 2.4 Statistical methods

A flow chart of the meta-analyses is shown in **Figure 1**, and one of the additional analyses is shown in **Supplementary Figure 1**.

#### 2.4.1 Study-level SNV-eGFR_creatinine_ association analysis in diabetes and subsequent meta-analysis

In each DM cohort and UKBB-T2D, associations between eGFR_creatinine_ and genetic variants were assessed assuming an additive genetic model. We used natural log-transformed eGFR_creatinine_ in a linear regression model (model 1) adjusted for traditional clinical and genetic factors, i.e. age, gender, and study-specific covariates (i.e., 0-10 principal components of population structure to account for population stratification). To control for potential bias on kidney function in the diabetes population, another model was further adjusted for HbA_1C_, systolic blood pressure (a proxy for medication with Angiotensin receptor blockers (ARBs) or Angiotensin-converting enzyme inhibitor (ACEi) frequently used in diabetes treatment) and diabetes duration (model 2). Some of the cohorts used summary statistics calculated prior to our query, so we allowed minor deviations in the included covariates (**Supplementary Table 3**). A list of software used for association analysis can be found in **Supplementary Table 1**. Each study dealt with missing data separately. Once all summary results were collected, we performed study-level quality control. Summary results were meta-analyzed using a fixed-effect inverse-variance method in the “Metagen” package in R (version 3.6.3). We report results in any diabetes mellitus subtype (denoted “combined”) and in T1D and T2D subsets. Significant heterogeneity (P_het_< 0.05) indicated variation across studies. Effect sizes (betas) are presented with 95% confidence intervals. We evaluate statistical significance at an FDR-corrected level of 0.05/4 = 0.0125 considering the number of tested SNVs.

#### 2.4.2 Additional analyses in UKBB populations with diabetes and non-diabetes

To explore the interplay between *CUBN*-variants and kidney-related traits in more detail, we did a range of additional linear regressions in the UKBB NDM and T2D groups. Further, we also applied a combined genetic risk score (GRS). We based the analyses on model 1 and model 3. The latter was very similar to model 2, in that it included adjustment for model 1 and SBP but not HbA_1c_ and diabetes duration. The last two adjustments were absent from this model because they are less relevant in non-diabetes. We applied the same models in DM and NDM to provide consistency. Individuals were excluded if they had missing data for any variable.

##### 2.4.2.1 SNV-eGFR_creatinine_ association analysis in the UKBB population without diabetes and replication in the deCODE study

We examined SNV-eGFR_creatinine_ associations in the UKBB NDM and T2D populations. It was advantageous to use the UKBB dataset here as it is a well-powered, phenotypically homogenous dataset (n ~ up to 370,000 individuals without diabetes). Since effects are based on natural log-transformed eGFR (trait) values, we also calculated the percental difference in mean, non-transformed eGFR per added effect allele for significant effects as follows: *%* *difference* =(*e* ^*beta*−1^)*100*%*. . Again, we evaluated statistical significance at an FDR-corrected level of 0.0125.

SNV-eGFR_creatinine_ associations identified in the UKBB NDM group were also examined in the Icelandic deCODE study (n_NDM_=127,090) applying model 3.

##### 2.4.2.2 Interaction with albuminuria

In order to examine whether the SNVs associated with eGFR_creatinine_ in an albuminuria-dependent fashion, we assessed albuminuria-SNV interactions in SNV-eGFR_creatinine_ regression models in individuals with T2D (n_T2D_ = 7,777) and without DM (n_NDM_ = 107,276) for whom continuous urine albumin levels were available (derived from the UKBB “microalbumin” field). The interaction term in the regression models included albuminuria groups as a factor defined from these albumin levels as follows: *i) normoalbuminuria:* =< 30 mg/L (n_DM_ = 5,566, n_NDM_ = 93,728), *ii) microalbuminuria:* 30-300 mg/L (incl. lower but not upper threshold, nN_DM_ = 1,954, n_NDM_ = 12,690), and *iii) macroalbuminuria:* >300 mg/L (incl. lower threshold, n_DM_ = 257, n_NDM_ = 858). We used regression models based on model 1 and 3 (i.e., *model 1*: ln(eGFR_creatinine_) ~ SNV + albuminuria group + age + sex + SNV*albuminuria group and *model 3*: model 1 + SBP). A significant p-value (< 0.05) for the SNV*albuminuria interaction term was considered evidence for interaction. Interaction analysis was done whenever primary SNV-eGFR_creatinine_ analyses were well-powered.

##### 2.4.2.3 Genetic risk score association with microalbuminuria and eGFR_creatinine_

We estimated an albuminuria genetic risk score (GRS) using the four albuminuria-associated *CUBN* missense SNVs. The GRS was generated for each study participant using the sum of individual SNV effect alleles in the UKBB dataset. We then examined the associations between GRS*_CUBN_* and continuous urine microalbumin levels (mg/L) and eGFR_creatinine._

##### 2.4.2.4 SNV vs. other kidney function-related traits in UKBB

We examined the associations between the study SNVs and 1) circulating serum Cystatin C levels (mg/L) and 2) the more recent eGFR_creatinine-cystatin C_ equation (30) that uses both serum creatinine and cystatin C levels and applies to all ethnicities.

#### 2.4.3 Power calculations

We used Quanto (version 1.2.4) (31) to calculate *post-hoc* power for main SNV-eGFR_creatinine_ associations in DM and NDM groups.

For all power calculations in Quanto, we: *a)* chose a continuous design for independent individuals; *b)* assumed a gene-only hypothesis; *c)* assumed an additive inheritance mode; and *d)* set the two-sided type I error-rate to 0.05.

For the remaining options in Quanto, we typed in information specific to each variant and population (**Supplementary Tables 13**-**14**): For each variant, we used allele frequencies of the effect allele; for meta-analyses, this was done as a range of calculations spanning the frequencies reported by individual cohorts. We used effect sizes obtained through DM and NDM SNV-eGFR_creatinine_ association analyses (main effect). Means and standard deviations of ln(eGFR_creatinine_) were derived from UKBB subsets. Unless otherwise specified, total DM sample sizes were used.

## 3 Results

### 3.1 Clinical characteristics

Up to 34,743 individuals with diabetes mellitus (type 1 diabetes (T1D), n ~ 3,588, or type 2 diabetes (T2D), n ~ 31,155) and up to 370,061 without diabetes participated in the current study (**Figure 1** and **Supplementary Figure 1**). Clinical characteristics of participating studies can be found in **Table 1** and **Supplementary Tables 4**–**7**.

**Table 1:** Clinical characteristics of participating studies.

Study name	DM type	Indi-viduals (N)	Males (N, %)	Age^##^ [years]	BMI [kg/m^2^]	eGFR_creatinine_ [ml/min/1.73 m^2^]	SBP [mmHg]	Diabetes duration [years]	Urinary albumin		
									AER [mg/24h]	UACR [mg/mmol]	ALB [mg/L]
AfterEU	T1D	854	492 (57.60)	43.67 (11.15)	24.23 (3.21)	89.48 (26.61)	139.22 (20.90)	28.02 (9.50)	29.00 (7.00 - 618.00)	NR	NR
UK-ROI	T1D	1,410	716 (50.80)	45.09 (11.35)	26.30 (4.40)	54.30 (30.00)	135.02 (20.80)	30.45 (9.70)	NA	NA	NA
GENESIS	T1D	1,324	700 (52.90)	41.37 (12.21)	22.21 (8.15)	80.87 (28.49)	129.41 (23.75)	24.91 (10.45)	9.00 (4.16-37.25)	NR	NR
DiaGene	T2D	1,886	1,011 (53.60)	65.24 (10.57)	30.47 (5.43)	78.33 (20.55)	141.83 (18.72)	10.09 (8.45)	NR	5.85 (30.45)	NR
Rotterdam	T2D	1,022	487 (47.70)	68.10 (9.70)	29.40 (4.80)	78.30 (16.40)	147.10 (21.70)	NA	NA	NA	NA
ANDIS	T2D	9,367	5,548 (59.22)	66.29 (13.29)	30.77 (5.70)	84.69 (30.92)	NA	8.07 (4.40)	NA	NA	NA
ExWas**	T2D	3,990	2,370 (59.30)	61.00 (8.50)	NA	79.00 (1.28)	NA	NA	NA	NA	NA
UKBB- T2D^#^	T2D	14,890	9,703 (65.10)	60.97 (6.28)	31.90 (5.70)	87.86 (15.73)	144.50 (18.20)	NA	NR	NR	16.00 (10.00-34.40)
UKBB-NDM^#^	NR*	370,061	166,976 (45.10)	56.73 (8.02)	27.10 (4.50)	90.81 (12.80)	139.90 (19.60)	NR	NR	NR	11.10 (8.30-18.10)

*Non-DM population. **The ExWas study comprises summary data from T2D individuals (discovery set). N, sample size; SD, standard deviation; BMI, Body-Mass Index; eGFR_creatinine_, estimated glomerular filtration rate based on the CKD-EPI_2012_ equation (non-transformed); SBP, Systolic blood pressure; AER, albumin excretion rate; IQR, Interquartile range; UACR, urinary albumin-creatinine ratio; UKBB, UK Biobank; ALB, continuous baseline urinary albumin level; T2D, Type 2 diabetes; DM, diabetes mellitus; NDM; non-DM; T1D, type 1 diabetes. NR, not relevant; NA, not available. ^#^The UK Biobank urinary albumin measures are based on n=7,777 in T2D and n=370,061 in the NDM group. ^##^The time point for age assessment is NA for Genesis. Age at recruitment was used in all other studies. Age, BMI, eGFR, and SBP have been deonted as mean (SD), while Urinary albumin measures have been denoted as median (IQR).

### 3.2 *CUBN* variants are not associated with eGFR_creatinine_ in a diabetes meta-analysis

The effect of *rs144360241* on eGFR_creatinine_ was studied in 32,904 individuals with diabetes. The variant was not available in UK-ROI (**Supplementary Figures 2**, **6**). All eight studies contributed to the 34,050 individuals analyzed for *rs45551835* (**Supplementary Figures 3** and **7**). The *rs141640975* variant was available for 32,993 individuals and was unavailable in UK-ROI (**Supplementary Figures 4**, **8**). The common variant, *rs1801239*, was available in all eight studies in 34,070 individuals (**Supplementary Figures 2**, **9**).

After meta-analysis, none of the four *CUBN* variants were significantly positively associated with eGFR_creatinine_ in the DM group, neither in the T1D or T2D subgroup [**Table 2** (Model 1) and **Table 3** (Model 2)]. However, the positive directionality of the effect for the T2D group was consistent with the directionality of effect for the combined group for all variants with non-zero effects. The T2D group carried the largest weight in the combined meta-analyses and UKBB carried the largest weight within the T2D group (**Supplementary Figures 2**–**5**). There was no evidence of heterogeneity across studies, except in model 2 for *rs45551835* and *rs1801239* (**Table 3**).

**Table 2:** meta-analysis of SNV-eGFR_creatinine_ summary data in diabetes mellitus and its subtypes (model 1).

Genetic variant (EA)	Diabetes type	N	Effect (Beta [95% CI])	P_HET_	P-value
rs144360241 (C)	T1D	2,177	-0.14 [-0.32; 0.05]	0.38	0.15
	T2D	30,727	0.01 [-0.01; 0.04]	0.62	0.40
	Combined DM	32,904	0.01 [-0.02; 0.04]	0.42	0.53
rs45551835 (A)	T1D	3,236	-0.02 [-0.13; 0.08]	0.34	0.69
	T2D	30,814	0.01 [0.00; 0.03]	0.08	0.09
	Combined DM	34,050	0.01 [0.00; 0.02]	0.15	0.10
rs141640975 (A)	T1D	2,177	0.16 [-0.11; 0.44]	0.75	0.25
	T2D	30,816	0.00 [-0.03; 0.03]	0.53	0.83
	Combined DM	32,993	0.01 [-0.02; 0.03]	0.60	0.73
rs1801239 (C)	T1D	3,236	-0.01 [-0.06; 0.03]	0.20	0.57
	T2D	30,834	0.00 [0.00; 0.01]	0.21	0.64
	Combined DM	34,070	0.00 [0.00; 0.01]	0.23	0.59

SNV, single nucleotide variant; eGFR_creatinine_, log-transformed estimated glomerular filtration rate based on the CKD-EPI_2012_ equation; EA, effect allele (i.e., minor allele); N, sample size; Beta, Beta coefficient; CI, confidence interval; P_Het_, P-value for heterogeneity across studies. P_Het_< 0.05 indicates variation; T1D: Type 1 diabetes; T2D: Type 2 diabetes; Combined DM: T1D and T2D combined.

**Table 3:** meta-analysis of SNV-eGFR_creatinine_ summary data in diabetes mellitus and its subtypes (model 2).

Genetic variant (EA)	Population	N	Effect (Beta [95% CI])	P_HET_	P-value
rs144360241 (C)	T1D	1,916	-0.12 [-0.32; 0.08]	0.26	0.25
	T2D	15,745	0.01 [-0.02; 0.04]	0.37	0.66
	Combined DM	17,661	0.00 [-0.02; 0.03]	0.32	0.78
rs45551835 (A)	T1D	2,712	-0.05 [-0.16; 0.07]	0.25	0.43
	T2D	15,724	0.01 [0.00; 0.03]	0.03*	0.14
	Combined DM	18,436	0.01 [0.00; 0.03]	0.05	0.18
rs141640975 (A)	T1D	1,916	0.10 [-0.17; 0.38]	0.4	0.46
	T2D	15,746	0.00 [-0.04; 0.05]	0.58	0.88
	Combined DM	17,662	0.01 [-0.04; 0.05]	0.67	0.8
rs1801239 (C)	T1D	2,712	0.00 [-0.04; 0.05]	0.53	0.94
	T2D	15,741	0.00 [-0.01; 0.01]	0.03*	0.77
	Combined DM	18,453	0.00 [-0.01; 0.01]	0.15	0.76

SNV, single nucleotide variant; eGFR_creatinine_, log-transformed estimated glomerular filtration rate based on the CKD-EPI_2012_ equation; EA, effect allele (i.e., minor allele); N, sample size; Beta, Beta coefficient; CI, confidence interval; P_Het_, P-value for heterogeneity across studies. P_Het_< 0.05 indicates variation; T1D: Type 1 diabetes; T2D: Type 2 diabetes; Combined DM: T1D and T2D combined.

### 3.3 *CUBN* variants are associated with higher eGFR_creatinine_ in non-diabetes

In UKBB-NDM, we observed larger eGFR_creatinine_-levels for minor alleles compared to major alleles for all four *CUBN* variants in both models, except for *rs1801239* in NDM, model 3 (**Table 4** and **Supplementary Table 8**): The effect and standard deviation of *rs144360241* was, for model 1 (model 3), 0.008 ± 0.002 (0.007 ± 0.002), corresponding to a difference of +0.8% (+0.7%) in mean eGFR_creatinine_ (ml/min/1.73 m^2^) for each additional copy of the affect allele, *C*. For *rs45551835*, the effect was 0.005 ± 0.001 (0.004 ± 0.001), corresponding to a difference of +0.5% (+0.4%) in mean eGFR_creatinine_ per copy of the *A*-allele. *rs141640975* had the largest effect size, 0.02 ± 0.003 (0.02 ± 0.003), corresponding to a +2.02% (+2.02%) difference in mean eGFR_creatinine_ for each additional *A*-allele. The common variant, *rs1801239*, had the smallest effect size of 0.001 ± 0.0005, corresponding to a +0.1% difference in eGFR_creatinine_ for each *C*-allele. We replicated the finding that *rs141640975* was significantly associated with higher eGFR_creatinine_ in non-diabetes in an Icelandic study (deCODE, n = 127,090, effect = 0.026, SE = 0.007, P_eGFR_creatinine_ = 7.7 × 10^-4^, model 3, **Supplementary Table 8**). None of the other SNVs were replicated (data not shown). Meta-analysis for the *rs141640975*-eGFR-association in the NDM studies (UKBB and deCODE) is depicted in **Supplementary Figure 10**.

**Table 4:** Summary results for SNV-eGFR_creatinine_ analyses in UKBB (model 1).

Genetic variant (EA)	EAF	Population **	N	Effect (Beta [SE])	P-value
rs144360241 (C)	0.004	NDM ***	369,832	0.008 (0.002)	0.0008*
	0.004	T2D ****	14,882	0.02 (0.02)	0.23
rs45551835 (A)	0.014	NDM ***	369,028	0.005 (0.001)	0.0004*
	0.014	T2D ****	14,860	0.01 (0.01)	0.13
rs141640975 (A)	0.003	NDM ***	369,987	0.02 (0.003)	2.2 × 10^-9^*
	0.003	T2D ****	14,885	-0.01 (0.02)	0.71
rs1801239 (C)	0.10	NDM ***	369,849	0.001 (0.0005)	0.006*
	0.10	T2D ****	14,880	0.00 (0.00)	0.42

SNV, single-nucleotide variant; eGFR_creatinine_, estimated glomerular filtration rate (natural log-transformed); EA, effect allele (i.e., minor allele); N, sample size; EAF, Effect allele frequency; Beta, Beta coefficient; SE, standard error; NDM, without Diabetes Mellitus; T2D, Type 2 diabetes. *Statistically significant (P< 0.05). ** For completeness, we also show the results for T2D, which were part of DM meta-analyses for model 1. *** out of total 370,061 individuals. **** out of total 14,892 individuals.

In UKBB-T2D, none of the variants had statistically significant associations with eGFR_creatinine_, although the effects of three of the variants (except *rs141640975*) were in the same direction as in NDM (**Table 4** and **Supplementary Table 8**).

### 3.4 Associations of *rs141640975* with eGFR_creatinine_ depend on albuminuria-status in non-diabetes

To examine whether the SNVs are associated with eGFR_creatinine_ in an albuminuria-dependent fashion, we included albuminuria*SNV interactions in two regression models. For the first model, we observed significant interaction for *rs141640975* in UKBB-NDM (P_interaction_ = 0.03, **Table 5**). This was also observed in the other model (P_interaction_ = 0.04, **Supplementary Table 9**). An interaction plot showed that for the eGFR-SNV-association, the effect on eGFR was even higher for more elevated albuminuria-levels (**Supplementary Figure 11**).

**Table 5:** Interaction with albuminuria in SNV-eGFR_creatinine_ analyses in UKBB (model 1).

Genetic variant (EA)	Population	N	P-value of interaction term^#^
rs144360241 (C)	NDM **	107,202	0.67
rs45551835 (A)	NDM **	106,964	0.88
rs141640975 (A)	NDM **	107,255	0.03*
rs1801239 (C)	NDM **	107,216	0.49

SNV, single-nucleotide variant; eGFR_creatinine_, estimated glomerular filtration rate (natural log-transformed); EA, effect allele (i.e., minor allele); N, sample size; NDM, without Diabetes Mellitus; *Statistically significant (P< 0.05). ** out of total 107,276 individuals with continuous urinary albumin levels. Albuminuria-SNV interaction was only tested when primary SNV-eGFRcreatinine associations were significant. # Interaction term is SNV*albuminuria groups (normo-, micro-, and macro albuminuria).

### 3.5 A *CUBN*-based GRS for albuminuria is associated with eGFR_creatinine_ in non-diabetes

We combined the four *CUBN* variants into a genetic risk score for albuminuria, verified its associations with continuous urine albumin levels and tested it against eGFR_creatinine_ in UKBB-T2D and UKBB-NDM. The GRS was associated with higher levels of both traits, except for eGFR in T2D (**Tables 6**, **7**).

**Table 6:** Summary results for GRS_CUBN_-eGFR_creatinine_ and -ALB analyses in UKBB (model 1).

Trait	Population	N	Effect (Beta [SE])	P-value
ALB	NDM **	106,814	0.05 (0.004)	2 × 10^-16^*
	T2D ***	7,741	0.08 (0.02)	0.004*
eGFR_creatinine_	NDM	368,521	0.002 (0.0004)	2 × 10^-6^*
	T2D	14,837	0.004 (0.003)	0.2

GRS_CUBN_, A genetic risk score based on a combination of the four CUBN genetic variants (minor alleles); N, sample size; Beta, Beta estimate; SE, standard error; ALB, continuous urinary albumin (mg/L, natural log-transformed); eGFR_creatinine_, estimated glomerular filtration rate (natural log-transformed); NDM, without Diabetes Mellitus; T2D, Type 2 diabetes. *Statistically significant (P< 0.05). ** out of total 107,276 individuals with continuous urinary albumin levels. *** out of total 7,777 individuals with continuous urinary albumin levels.

**Table 7:** Summary results for GRS_CUBN_-eGFR_creatinine_ and -ALB analyses in UKBB (model 3).

Trait	Population	N	Effect (Beta [SE])	P-value
ALB	NDM **	99,180	0.05 (0.004)	2 × 10^-16^*
	T2D ***	7,182	0.08 (0.02)	3 × 10^-4^ *
eGFR_creatinine_	NDM	343,988	0.002 (0.0004)	2 × 10^-5^*
	T2D	13,828	0.005 (0.003)	0.1

GRS_CUBN_, A genetic risk score based on a combination of the four CUBN genetic variants (minor alleles); N, sample size; Beta, Beta estimate; SE, standard error; ALB, continuous urinary albumin (mg/L, natural log-transformed); eGFR_creatinine_, estimated glomerular filtration rate (natural log-transformed); NDM, without Diabetes Mellitus; T2D, Type 2 diabetes. *Statistically significant (P< 0.05). ** out of total 107,276 individuals with continuous urinary albumin levels. *** out of total 7,777 individuals with continuous urinary albumin levels.

### 3.6 *rs141640975* is associated with additional markers of kidney function in non-diabetes

We examined the associations between the study SNVs and two additional markers of kidney function. The SNV *rs141640975* was associated with higher levels of eGFR_creatine-cystatin C_ [a more recent ethnicity-independent GFR-estimator (28)] and lower levels of cystatin C, both observed in NDM (**Supplementary Tables 10**–**12**). The eGFR_creatinine-cystatin C_ association of *rs144360241* was borderline significant in NDM.

### 3.7 Estimated power

#### 3.7.1 Meta-analysis (diabetes mellitus)

Given the ranges of EAFs obtained from individual studies participating in meta-analyses, we reached a power level of 35-43% for *rs45551835*, 16-23% for *rs1444360241*, and 9-21% for *rs141640975* in the DM group (**Supplementary Table 14**). Effect sizes were assumed from the individual meta-analysis eGFR_creatinine_-associations of each SNV. We did not calculate power for *rs1801239* as the effect in the DM meta-analysis was 0.0.

#### 3.7.2 Association of SNVs with eGFR (UKBB population without diabetes)

In NDM, the power for main eGFR_creatinine_ analyses was between 70-99% for the four variants (**Supplementary Table 15**).

## 4 Discussion

Recently, we demonstrated that individuals carrying the minor allele of the *CUBN* missense variant *rs141640975* had higher albuminuria-levels than non-carriers. The effect of this variant was stronger in individuals with diabetes (DM) compared to those without diabetes (NDM) (11). In continuation of these findings, Bedin et al. (8) performed secondary lookups for *CUBN*-variants in the CKDGen eGFR GWAS study population, reporting that missense variants in *CUBN* may also be associated with higher levels of eGFR in the general population. Our current large-scale study aimed to examine the effect of minor alleles of three rare *CUBN* missense variants (*rs144360241 (c.6469A>G; p.Asn2157Asp)*, *rs45551835 (c.8741C>T; p.Ala2914Val)* and *rs141640975* (*c.5069C>T; p.Ala1690Val*)) and one common variant (*rs1801239 (c.8950A>G; p.Ile2984Val)*) on eGFR_creatinine_ levels separately in people with and without diabetes (n_DM_ ~ 34,000 individuals, n_NDM_ ~ 370,000 individuals), including stratification for diabetes-type and supplemented by tests on circulating cystatin C levels, the recently updated eGFR-equation based on creatinine and cystatin C (30), and aggregate-variant tests. We were able to replicate the association between creatinine-based eGFR and *rs141640975* in NDM and report new insightful connections with the alternative measures of kidney function for all four SNVs.

Previously, a borderline association between *rs45551835* and higher eGFR-levels has been reported in a smaller type 2 diabetes (T2D) population from Denmark (8, 11), a finding which we could not replicate in our meta-analysis of up to 34,432 individuals with diabetes and its subtypes. Like the initial study (8), we could not establish a link between eGFR and the three other variants within the diabetes group. As for *rs45551835*, it was surprising to be unable to replicate the earlier findings as the current study has a larger sample size compared to earlier efforts. Our *post-hoc* power assessment indicated that insufficient power might be at play, even with a larger sample size for the diabetes group (8). We also speculated whether the apparent lack of association between *CUBN* and eGFR in our diabetes meta-analysis could be due to use of Angiotensin receptor blockers (ARBs) or Angiotensin-converting enzyme inhibitor (ACEi) medication which is frequently used in diabetes treatment. As part of our sensitivity analyses, we included models adjusted for systolic blood pressure (a proxy for such medication) and did not find evidence that this could explain why no association was found in the diabetes group. Another reason could be the allele frequency of the variants may differ between Danish and UK populations. We need further validation in well-powered populations to confirm the relationship between the *rs45551835* and eGFR in diabetes, especially in T2D. In case of a true lack of association, *CUBN* may be associated with higher levels of urine albumin (11) with no pleiotropic effect to eGFR in this population.

We proceeded to single- and aggregate-variant analyses in the UK Biobank (UKBB), shifting focus to non-diabetes populations. For all four *CUBN* variants, we report significantly higher eGFR_creatinine_-levels in individuals without diabetes harboring more copies of the minor alleles compared to individuals with fewer or no copies of the minor alleles in the same group. For *rs141640975*, we observed the strongest association with eGFR_creatinine_ (*P* = 2.2 × 10^-9^) with replication in the Icelandic study (deCODE, P = 7.7 × 10^-4^), confirming what has previously been observed for this SNV in NDM (8) – but also a significant interaction between the SNV and albuminuria stages (*P_INT_<* 0.05). Taken together with the already known associations of the minor alleles with higher albuminuria (11), this not only demonstrates genetic pleiotropy of *CUBN* for albuminuria and eGFR in non-diabetes but also implies that these two associations are intertwined for this SNV, where the effect on eGFR is even higher for more elevated albuminuria-levels. Here, *CUBN* demonstrates a classic genetic pleiotropy phenomenon where a DNA variant influences multiple traits, usually in the same domain with concordant or sometimes discordant effects as observed earlier in complex disorders (32). Further validation of independent biological or related causal effects might be required in additional follow up studies.

This finding is unusual as there is no obvious clinical or pathophysiological explanation for such an albuminuria-eGFR pattern in the context of non-diabetes. It has been suggested that the tubular albuminuria observed in presence of C-terminal variants in *CUBN* has a benign or even slightly protective effect on kidney function in chronic kidney disease if glomerular albuminuria is also present (8, 33, 34). Another recent study on chronic isolated proteinuria suggests that different C-terminal *CUBN* variants uncouple proteinuria from glomerular filtration barrier through declined cubilin expression accompanied by aberrant amnionless (AMN) localization in renal tubules. AMN is part of the receptor complex (along with cubilin and megalin) necessary for tubular reabsorption of albumin. This is suggested to create a benign condition, not requiring any further proteinuria lowering treatment (35). In non-diabetes, where the population can be assumed to consist mostly of healthy individuals, a concept of such protectiveness is less relevant. However, it is possible that an undetected subpopulation with relevant comorbidities exists in the non-diabetes group.

Our *CUBN* aggregate-variant method – which was defined as a genetic risk score (GRS) combining the four variants – showed that a higher number of C-terminal *CUBN* risk alleles is associated with higher urine albumin and eGFR_creatinine_ levels and confirms both the single-variant association with higher urine albumin levels reported previously in diabetes and non-diabetes (11, 14), and the consistency of the overall effects on urine albumin levels being greater in diabetes compared to non-diabetes (10, 11). Through GRS*_CUBN_*, we also saw that a higher number of minor alleles across the four variants was associated with higher eGFR_creatinine_-levels in the UKBB population without diabetes, which is in line with our single-variant findings and the previous findings for *rs45551835* (8). Using aggregate-variant methods is an optimal way to examine combined genetic effects and has been used extensively for polygenic traits (13, 36). Using GRS is highly relevant here as three of the four variants are rare and mostly present as heterozygous variants in our populations. This might substantiate with some additional power to detect effects and adds further certainty to the presence of a *CUBN*-eGFR relationship in non-diabetes. Nevertheless, we still do not find an association with eGFR in T2D, even when the variants are combined in a GRS.

Finally, we examined the association between the study SNVs and two alternative markers of kidney function. In non-diabetes, the minor alleles of *rs141640975* and *rs144360241* were associated with higher levels of eGFR_creatinine-cystatin C_. This measure was estimated using a recent update to the equation, CKD-EPI_2021_, which does not include ethnicity and is a more precise indicator of kidney function in comparison to the CKD-EPI_creatinine(2012)_ equation which is based only on creatinine. Our results using the conventional eGFR_creatinine_ equation are concordant with our results from the updated equation in terms of directionality of effect and with our finding that *rs141640975* is associated with lower cystatin C levels, which is another indicator of kidney function. It should be noted, though, that considering **Table 1** and **Supplementary Tables 4**–**6**, the 0.1% – 2.02% higher mean eGFR we report for each minor allele is modest and may reflect that individual harboring these genetic variants have normal kidney function rather than a better kidney function.

A strength of our study is the restriction to specifically diabetes- and non-diabetes-only subgroups so that effects from mixed diabetes-status are minimized. Heterogeneity is likely to be present in meta-analyses of a diverse set of cohorts originally used for different research purposes. Indeed, some of the cohorts included in our meta-analyses differ regarding available covariates and/or kidney disease status. However, we did not observe heterogeneity in our meta-analyses. In addition to this, we could minimize heterogeneity in the remainder of our analyses by using data from the UKBB, which is a nationally representative cohort facilitating application of identical phenotype definitions across subgroups. Another strength is the broad spectrum of additional analyses that we explored in the UKBB population to nuance our findings on the relationship between eGFR and *CUBN*. The judicious use of UKBB leveraging individual-level genotype information to investigate interaction-analyses based on albuminuria groupings is a great strength of the current study, especially for rare variants.

A major limitation is that we did not have sufficient statistical power for our meta-analyses in the diabetes group due to the limited availability of suitable datasets. Consequently, interpretations of T2D findings should not be overstated and we thus could not demonstrate, nor disprove, the presence of a *CUBN*-eGFR relationship in this population. Although we demonstrate that C-terminal missense variants in *CUBN* are associated with different measures of normal (or even higher) kidney function in non-diabetes, we emphasize that the current study is insufficient to establish causality. Finally, using multiple-testing-corrected significance thresholds might be too conservative when testing a very small number of variants from the same locus as it may remove true associations. In genome-wide studies, a conservative threshold of 5 × 10^−8^ is generally agreed upon for novel associations. There is less consensus on when and how to appropriately apply multiple testing correction in smaller-scale genetic studies dealing with a mixture of new and known associations. Nevertheless, we deemed that it would be fair to apply FDR-correction of the significance threshold to our primary analyses in DM and NDM.

In conclusion, the current study identifies the existence of pleiotropic genetic effects of *CUBN* on two facets of kidney function – albuminuria and eGFR – by reporting SNV-eGFR associations in a large study population without diabetes. The interaction between *rs141640975* and albuminuria-status on eGFR_creatinine_ in this population and its associations with lower cystatin C and higher levels of eGFR_creatinine-cystatin C_ expands our knowledge of these variants in relation to measures of kidney function. The demonstration of a *CUBN*-focused GRS in relation to albuminuria and eGFR_creatinine_ further suggests an important role of *CUBN*-variants in the future personalization of chronic kidney disease management.

## Data availability statement

The original contributions presented in the study are included in the article/**Supplementary Material**. Further inquiries can be directed to the corresponding author.

## Ethics statement

The studies involving human participants were reviewed and approved by Ethical approval has previously been obtained locally for each individual study. The patients/participants provided their written informed consent to participate in this study.

## Author contributions

NU, MS, PR, and TA contributed to conception and design of the study. NU wrote the first draft of the manuscript.NU, MC-G, CH, AN, AO, SS, D-AT, EA, MH, AM, EJS, MS, PR, and TA contributed to manuscript revision. D-AT, VS, KS, EA, MH, AM, PR and TA acquired data. NU, FA, MC-G, CH, AN, SS, LS, D-AT, and TA performed statistical analysis. NU, MC-G, LS, D-AT, AM, MS, PR, and TA contributed to interpretation of data. TA and MS acquired funding and TA administered this project. PR and TA supervised the project. LC and MG had other roles. All authors contributed to the article and approved the submitted version.
