# Supplementary material for: Four missense genetic variants in CUBN are associated with higher levels of eGFR in non-diabetes but not in diabetes mellitus or its subtypes: A genetic association study in Europeans
Source: Front Endocrinol (Lausanne). 2023 Feb 28;14:1081741. doi: 10.3389/fendo.2023.1081741 (PMC10011651; doi:10.3389/fendo.2023.1081741)
Supplement: Supplementary file 2 [file DataSheet_1.docx]

**1. Supplemental text**

**Brief descriptions of participating cohorts**

***1.1 AfterEU***

Adult Caucasian patients with type 1 diabetes (T1D) attending the outpatient clinic at the Steno Diabetes Center Copenhagen between years 1993 to 2000 were invited to participate in a study of genetic risk factors for the development of diabetic complications. 73% of all agreed to participate. T1D was considered present if the age at onset of diabetes was < 35 years and the time to definite insulin therapy was < 1 year. In total, the cohort comprises 900 T1D individuals: 458 individuals with persistent albuminuria (> 300 mg/24 h) in two out of three consecutive measurements, the presence of retinopathy, and the absence of other kidney or urinary tract disease and 442 individuals with persistent normoalbuminuria (< 30 mg/24 h) after > 15 years of type 1 diabetes (1–4).

***1.2 Rotterdam***

This study is embedded within the framework of the Rotterdam Study, a prospective population-based cohort among people ≥ 40 years living in the well-defined Ommoord district of Rotterdam, the Netherlands. The complete design and rationale behind the Rotterdam Study are described in a separate publication (5). This study included participants from the third examination of the first cohort (1997-1999), the first examination of the second cohort (2000-2001), and the first examination of the third cohort (2006-2008). We included participants if they had information on genetic variants, prevalent diabetes status and eGFR measurements (n=8553) (5).

***1.3 DiaGene***

The overall aim of the DiaGene study is to unravel the etiology of type 2 diabetes and its complications, by identifying risk factors e.g. genomic, glycomic and lipidomic factors.

The design of the DiaGene study has been reported elsewhere (6). Briefly, the DiaGene study is an all lines of healthcare prospective case-control study collected in and around the city Eindhoven in the Netherlands. All hospitals in this area participated, as well as the center for primary care diagnostics.

***1.4 UK-ROI***

This UK-ROI collection consists of samples derived from the Republic of Ireland (ROI) and the United Kingdom (Warren 3 and Genetics of Kidneys in Diabetes UK, UK GoKinD), as part of the Genetics of Nephropathy: an International Effort (GENIE) and the Diabetic Nephropathy Collaborative Research Initiative (DNCRI) (7). This study population included 1,804 white individuals diagnosed with type 1 diabetes mellitus before 31 years of age, with parents and grandparents born in the UK and Ireland and full details of these have been previously described (7).

***1.5 Genesis***

Patients from the GENESIS (genes nephropathy and sib pair study) and the GENEDIAB (Génétique de la Néphropathie Diabétique) and non-duplicate consecutively-recruited type 1 diabetes participants from Corbeil, Paris St Louis, Nantes, Toulouse and Poitiers were considered (8). They were individuals of European ethnicity with type 1 diabetes (T1D). American Diabetes Association diagnostic criteria, as defined in 1997, were used for diabetes classification. T1D was defined by the age at diabetes onset before 35 years. All T1Ds participating in the GENESIS study had retinopathy and a diabetes duration of more than 15 years (9), while participants in the GENEDIAB study had severe diabetic retinopathy (severe non-proliferative with retinal panphotocoagulation and/or proliferative retinopathy) while other participants were not selected on any specific criterion (10). A total of 1,324 individuals with eGFR measures and genotype data participated in the current study.

***1.6 ANDIS***

The ANDIS cohort includes 14,625 incident cases of diabetes recruited within Scania (Skåne) County in southern Sweden (1,200,000 inhabitants), during the period January 2008 until November 2016. During this period 177 healthcare clinics registered patients, aged 0–96 years, within a median of 40 days (interquartile range = 12–99) after diagnosis. Individuals aged 18 and older were included in the analysis. Patients with known secondary diabetes were excluded (11).

***1.7 UK Biobank***

The UK Biobank study was accessed in reference to application ID number 32683 and 71699. We defined two study populations - type 2 diabetes (UKBB-T2D) and non-diabetes (UKBB-NDM) - using previously described definitions (12,13) adapted to availability of data fields in the application (briefly summarized below), e.g. excluding ICD10 codes. The approach we used to calculate eGFR in UKBB is also described.

***1.7.1 Type 2 diabetes (UKBB-T2D)***

Individuals having Type 2 Diabetes were defined using the following Data Fields and criteria from the UKBB:

1. All answering “Yes” to “Diabetes diagnosed by doctor” (Field ID: 2443). Reports of either type 2 diabetes or generic diabetes at the interview.
2. Age diabetes diagnosed (Field ID: 2976) >35 years. Reported age at diagnosis over 35 years to limit the participants with slow progressing autoimmune or monogenic diabetes forms.
3. All individuals who did not start insulin within one year diagnosis of diabetes. All saying “No” to “Started insulin within one year diagnosis of diabetes” (Field ID: 2986).

We excluded individuals who:

1. Did not report an age of diagnosis
2. Responded “Yes” to “Gestational diabetes only” (Field ID: 4041).

***1.7.2 Non-diabetes (UKBB-NDM)***

We considered the non-diabetes group to be individuals who:

1. had never been diagnosed with diabetes
2. answered “No” to “Diabetes diagnosed by doctor” (Field ID: 2443).

***1.7.3 Phenotype/trait details***

The estimated glomerular filtration rate (eGFR_creatinine_) was calculated using Chronic Kidney Disease Epidemiology Collaboration (CKD-EPI_creatinine(2012)_) equation (14). In the UKBB, we used the following as part of the baseline characteristics: Blood Creatinine levels (Umol/L, Field ID: 30700), Genetic ethnic grouping (Field ID: 22006), age (Field ID: 21022) and sex (Field ID: 31). Blood creatinine levels were converted from Umol/L to mg/dl by a multiplication factor of 0.0113 (Umol/L x 0.0113 = mg/dl).

***1.8 deCODE (NDM)***

The deCODE consist of Icelandic individuals who have participated in genetic studies at deCODE genetics. The details of this study and phenotyping have been described previously (15). Non diabetes was classified based on absence of any form of diabetes based on ICD10. The estimated glomerular filtration rate (eGFR_creatinine_) was calculated using Chronic Kidney Disease Epidemiology Collaboration (CKD-EPI_creatinine(2012)_) equation (14).

**2. Supplementary figures**

**Supplementary figure 1:** flow chart of additional analyses in UKBB.


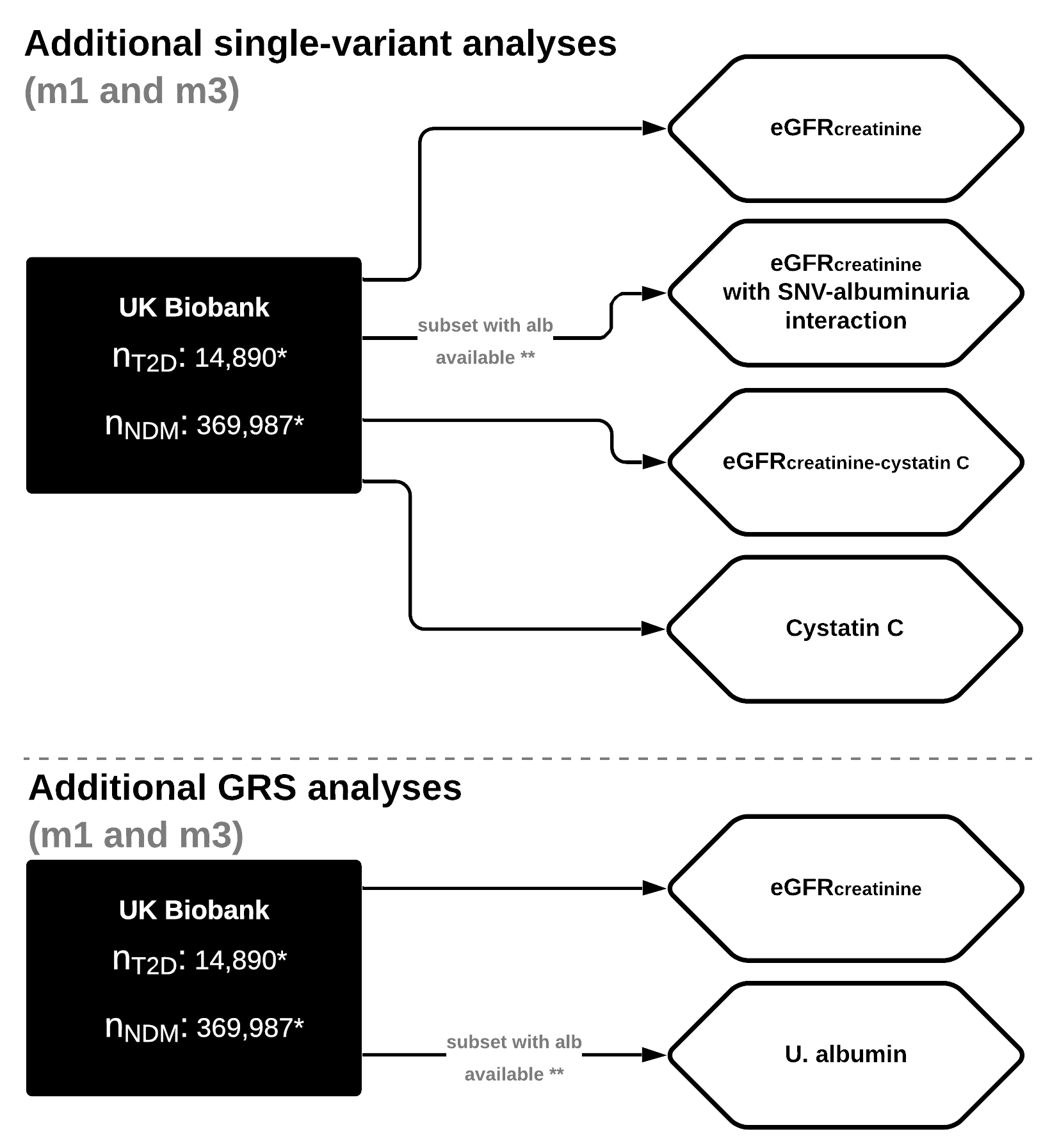


* Sample sizes (n) reflect the number of individuals available for *rs141640975*, model 1. ** The subsets are based on the n = 7,777 in UKBB-T2D and n = 107,276 in UKBB-NDM where urinary albumin measures were available. **m1:** model 1 (eGFR_creatinine_ ~ genotype + sex + age); **m3:** model 3 (m1 + SBP); **PCs:** Principal components of population structure; **SBP:** Systolic blood pressure. **T2D:** Type 2 diabetes; **NDM:** without diabetes; **eGFR_creatinine_:** Estimated glomerular filtration rate, natural log-transformed; **SNV:** single nucleotide variant; **eGFR_creatinine-cystatin C_:** Estimated glomerular filtration rate, natural log-transformed, calculated with a recent equation; **U. albumin**: continuous urinary albumin levels (mg/L), natural log-transformed; **GRS:** genetic risk score.

**Supplementary figure 2: Forest plot of associations of rs144360241 with eGFR_creatinine_ (model 1)_._**


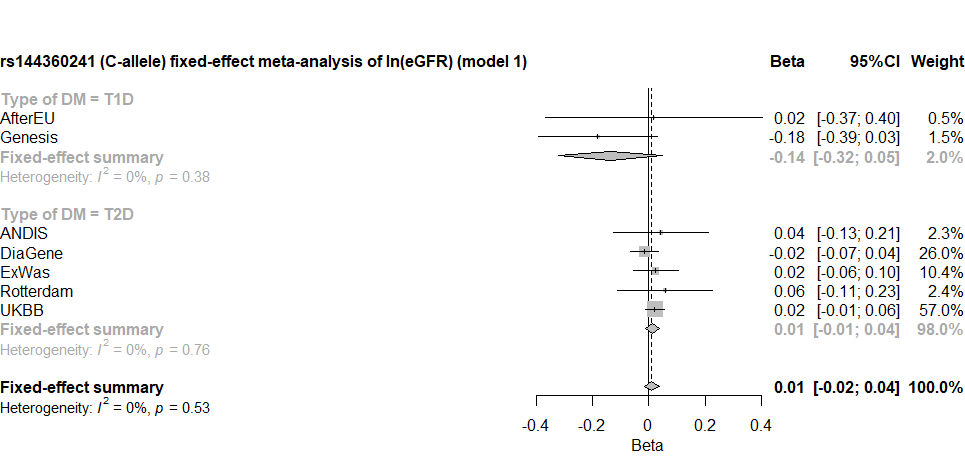


**Model 1:** eGFR_creatinine_ ~ genotype + sex + age + 0-10 PCs; **PC:** principal component of population structure; **DM:** diabetes mellitus; **T1D:** type 1 diabetes; **T2D:** type 2 diabetes; **ln(eGFR):** estimated glomerular filtration rate (natural log-transformed); **I^2^**: heterogeneity statistic; **p:** p-value of heterogeneity (i.e., p_HET_). Significant heterogeneity (P_het_ < 0.05) indicates variation across studies; **Beta:** beta value; **CI:** confidence interval.

**Supplementary figure 3: Forest plot of associations of rs45551835 with eGFR_creatinine_ (model 1)_._**

**
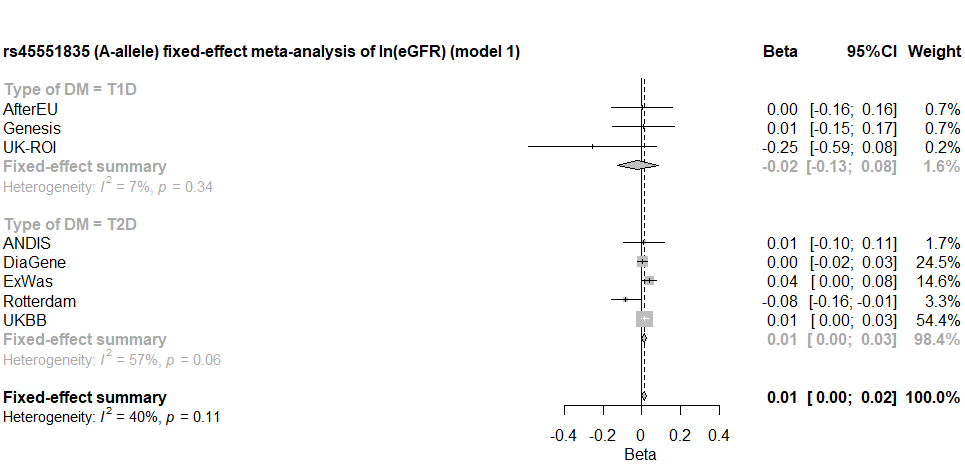
**

**Model 1:** eGFR_creatinine_ ~ genotype + sex + age + 0-10 PCs; **PC:** principal component of population structure; **DM:** diabetes mellitus; **T1D:** type 1 diabetes; **T2D:** type 2 diabetes; **ln(eGFR):** estimated glomerular filtration rate (natural log-transformed); **I^2^**: heterogeneity statistic; **p:** p-value of heterogeneity (i.e., p_HET_). Significant heterogeneity (P_het_ < 0.05) indicates variation across studies; **Beta:** beta value; **CI:** confidence interval.

**Supplementary figure 4: Forest plot of associations of rs141940975 with eGFR_creatinine_ (model 1)_._**

**
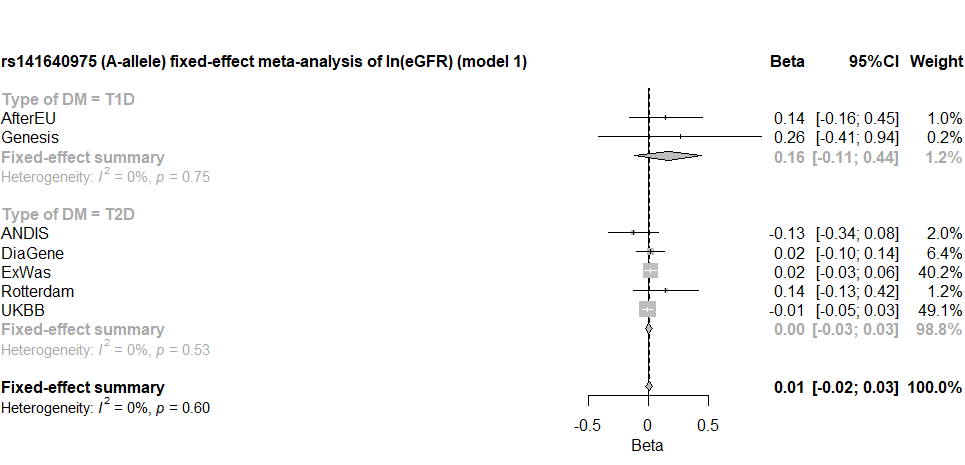
**

**Model 1:** eGFR_creatinine_ ~ genotype + sex + age + 0-10 PCs; **PC:** principal component of population structure; **DM:** diabetes mellitus; **T1D:** type 1 diabetes; **T2D:** type 2 diabetes; **ln(eGFR):** estimated glomerular filtration rate (natural log-transformed); **I^2^**: heterogeneity statistic; **p:** p-value of heterogeneity (i.e., p_HET_). Significant heterogeneity (P_het_ < 0.05) indicates variation across studies; **Beta:** beta value; **CI:** confidence interval.

**Supplementary figure 5: Forest plot of associations of rs1801239 with eGFR_creatinine_ (model 1)_._**


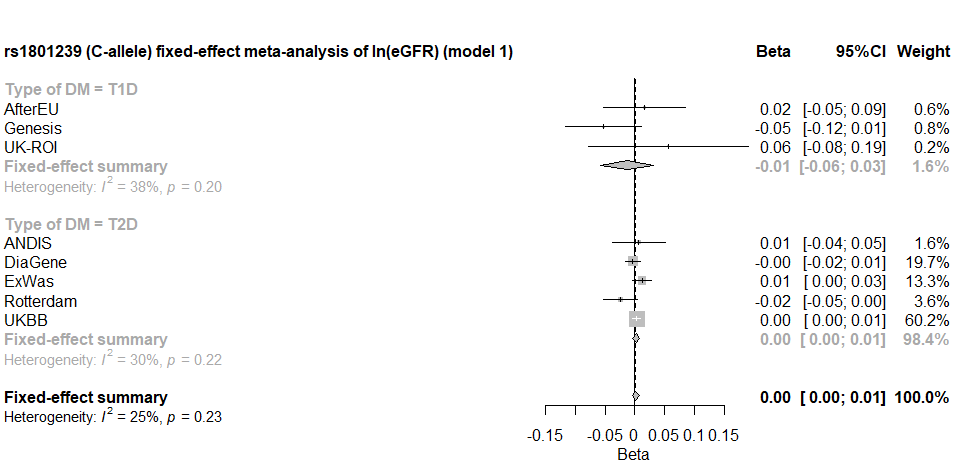


**Model 1:** eGFR_creatinine_ ~ genotype + sex + age + 0-10 PCs; **PC:** principal component of population structure; **DM:** diabetes mellitus; **T1D:** type 1 diabetes; **T2D:** type 2 diabetes; **ln(eGFR):** estimated glomerular filtration rate (natural log-transformed); **I^2^**: heterogeneity statistic; **p:** p-value of heterogeneity (i.e., p_HET_). Significant heterogeneity (P_het_ < 0.05) indicates variation across studies; **Beta:** beta value; **CI:** confidence interval.

**Supplementary figure 6: Forest plot of associations of rs144360241 with eGFR_creatinine_ (model 2)_._**

**
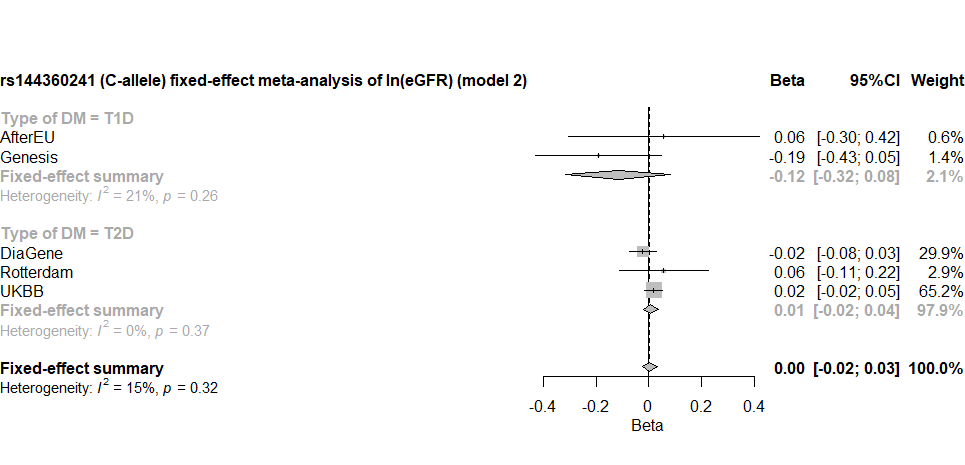
**

**Model 2:** eGFR_creatinine_ ~ genotype + sex + age + HbA_1C_ + SBP + DM duration + 0-10 PCs; **HbA_1C_:** hemoglobin A_1C_; **SBP:** systolic blood pressure; **PC:** principal component of population structure; **DM:** diabetes mellitus; **T1D:** type 1 diabetes; **T2D:** type 2 diabetes; **ln(eGFR):** estimated glomerular filtration rate (natural log-transformed); **I^2^**: heterogeneity statistic; **p:** p-value of heterogeneity (i.e., p_HET_). Significant heterogeneity (P_het_ < 0.05) indicates variation across studies; **Beta:** beta value; **CI:** confidence interval.

**Supplementary figure 7: Forest plot of associations of rs45551835 with eGFR_creatinine_ (model 2)_._**

**
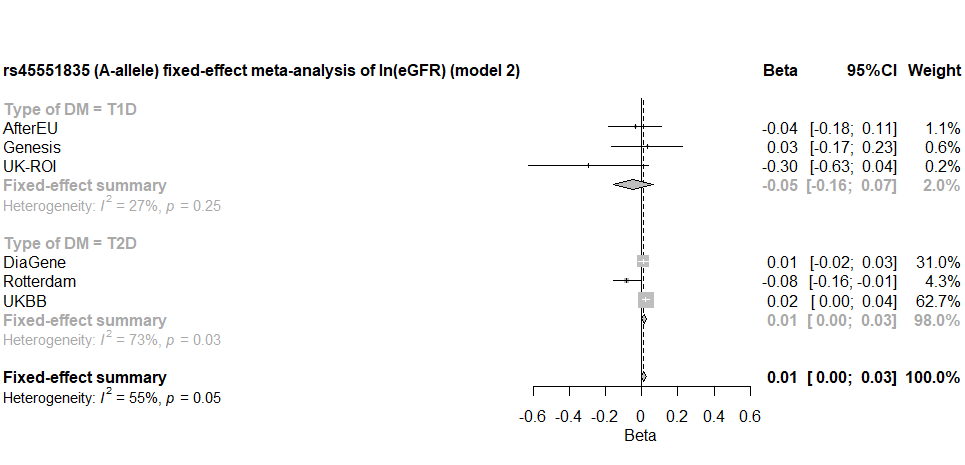
**

**Model 2:** eGFR_creatinine_ ~ genotype + sex + age + HbA_1C_ + SBP + DM duration + 0-10 PCs; **HbA_1C_:** hemoglobin A_1C_; **SBP:** systolic blood pressure; **PC:** principal component of population structure; **DM:** diabetes mellitus; **T1D:** type 1 diabetes; **T2D:** type 2 diabetes; **ln(eGFR):** estimated glomerular filtration rate (natural log-transformed); **I^2^**: heterogeneity statistic; **p:** p-value of heterogeneity (i.e., p_HET_). Significant heterogeneity (P_het_ < 0.05) indicates variation across studies; **Beta:** beta value; **CI:** confidence interval.

**Supplementary figure 8: Forest plot of associations of rs141940975 with eGFR_creatinine_ (model 2)_._**

**
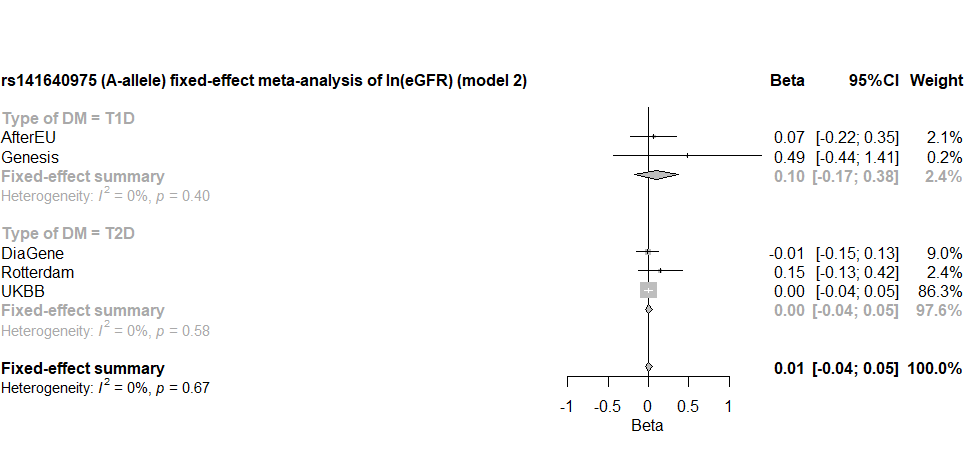
**

**Model 2:** eGFR_creatinine_ ~ genotype + sex + age + HbA_1C_ + SBP + DM duration + 0-10 PCs; **HbA_1C_:** hemoglobin A_1C_; **SBP:** systolic blood pressure; **PC:** principal component of population structure; **DM:** diabetes mellitus; **T1D:** type 1 diabetes; **T2D:** type 2 diabetes; **ln(eGFR):** estimated glomerular filtration rate (natural log-transformed); **I^2^**: heterogeneity statistic; **p:** p-value of heterogeneity (i.e., p_HET_). Significant heterogeneity (P_het_ < 0.05) indicates variation across studies; **Beta:** beta value; **CI:** confidence interval.

**Supplementary figure 9: Forest plot of associations of rs1801239 with eGFR_creatinine_ (model 2)_._**

**
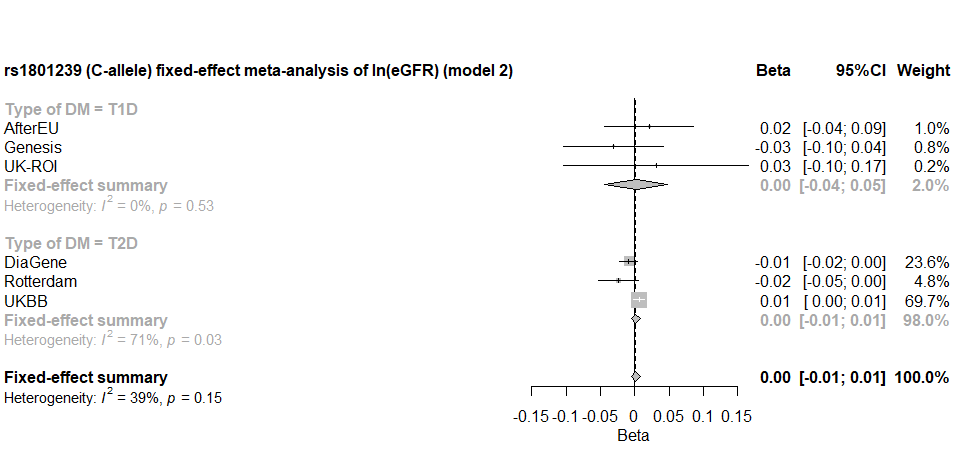
**

**Model 2:** eGFR_creatinine_ ~ genotype + sex + age + HbA_1C_ + SBP + DM duration + 0-10 PCs; **HbA_1C_:** hemoglobin A_1C_; **SBP:** systolic blood pressure; **PC:** principal component of population structure; **DM:** diabetes mellitus; **T1D:** type 1 diabetes; **T2D:** type 2 diabetes; **ln(eGFR):** estimated glomerular filtration rate (natural log-transformed); **I^2^**: heterogeneity statistic; **p:** p-value of heterogeneity (i.e., p_HET_). Significant heterogeneity (P_het_ < 0.05) indicates variation across studies; **Beta:** beta value; **CI:** confidence interval.

**Supplementary figure 10: Forest plot of associations of eGFR_creatinine_ (lneGFR) with rs141940975 in NDM cohorts (model 3)_._**

**_
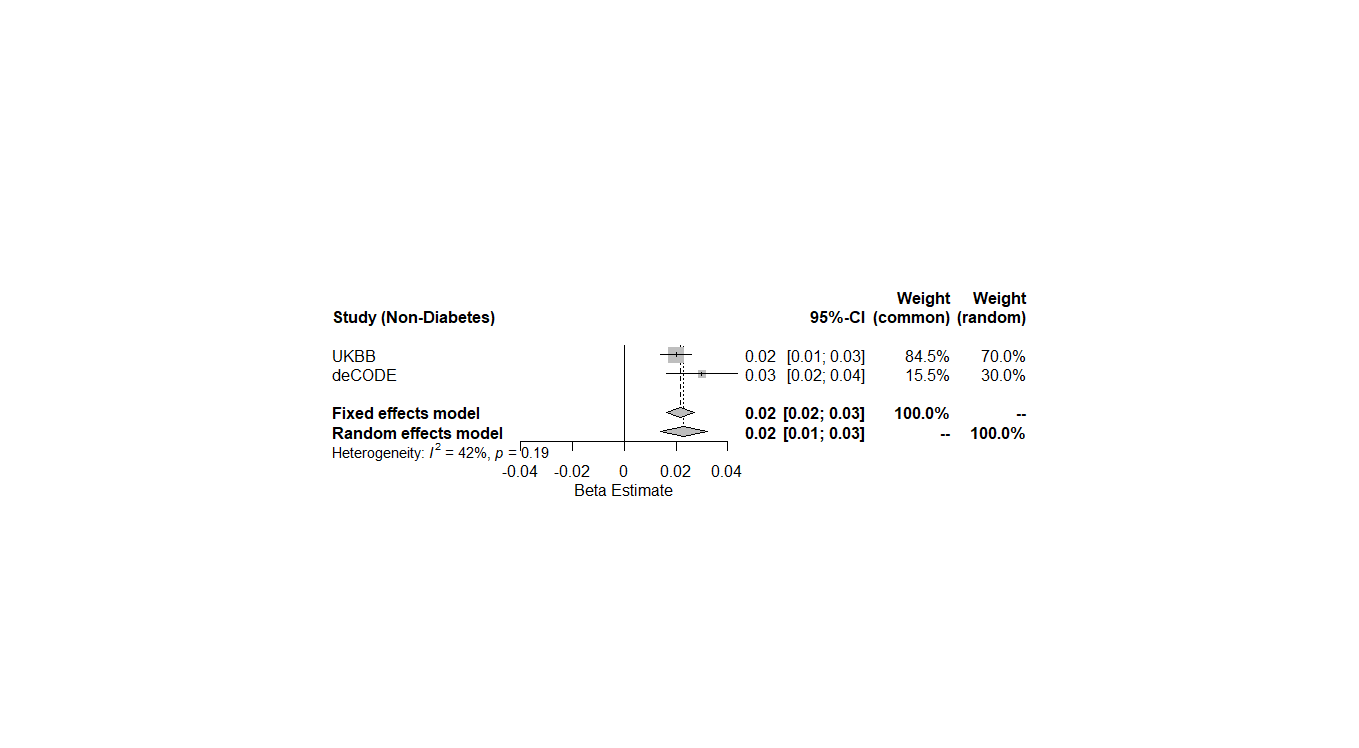
_**

**Model 3:** eGFR_creatinine_ ~ genotype + sex + age + SBP; **SBP:** systolic blood pressure; **NDM:** Non diabetes mellitus; **ln(eGFR):** estimated glomerular filtration rate (natural log-transformed); **I^2^**: heterogeneity statistic; **p:** p-value of heterogeneity (i.e., p_HET_). Significant heterogeneity (P_het_ < 0.05) indicates variation across studies; **Beta:** beta value; **CI:** confidence interval; **UKBB:** UK Biobank.

**Supplementary figure 11: Interaction plot of albuminuria level on SNV-eGFR_creatinine_ for rs141940975 in NDM.**


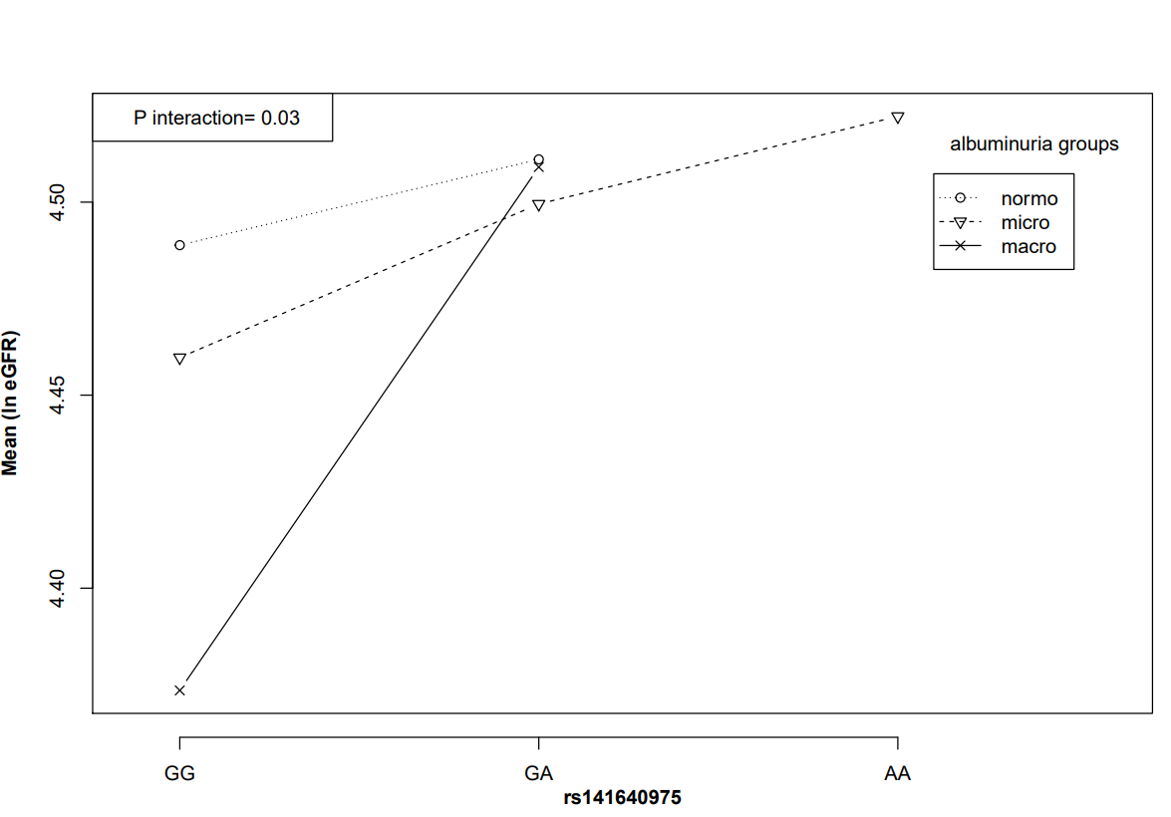


The y-axis indicates individuals with 0 (GG), 1 (GA), or 2 (AA) effect alleles where A is the effect allele and G is the non-effect allele. **SNV:** Single-nucleotide variant; **eGFR_creatinine_:** estimated glomerular filtration rate (natural log-transformed); **NDM**: population in UK Biobank without diabetes; **normo:** normoalbuminuria; **micro:** microalbuminuria; **macro:** macroalbuminuria; **P_interaction:** P-value for the SNV*albuminuria-group interaction (model 1).

**3. References**

1. Charmet R, Duffy S, Keshavarzi S, Gyorgy B, Marre M, Rossing P, et al. Novel risk genes identified in a genome-wide association study for coronary artery disease in patients with type 1 diabetes. Cardiovasc Diabetol. 2018;17(1):61.

2. Dahlström EH, Saksi J, Forsblom C, Uglebjerg N, Mars N, Thorn LM, et al. The Low-Expression Variant of FABP4 Is Associated With Cardiovascular Disease in Type 1 Diabetes . Diabetes. 2021;70(10):2391–401.

3. Winther SA, Øllgaard JC, Tofte N, Tarnow L, Wang Z, Ahluwalia TS, et al. Utility of Plasma Concentration of Trimethylamine N-Oxide in Predicting Cardiovascular and Renal Complications in Individuals With Type 1 Diabetes. Diabetes Care. 2019 Aug;42(8):1512–20.

4. Lajer M, Jorsal A, Tarnow L, Parving HH, Rossing P. Plasma growth differentiation factor-15 independently predicts all-cause and cardiovascular mortality as well as deterioration of kidney function in type 1 diabetic patients with nephropathy. Diabetes Care. 2010;33(7):1567–72.

5. Ikram MA, Brusselle G, Ghanbari M, Goedegebure A, Ikram MK, Kavousi M, et al. Objectives, design and main findings until 2020 from the Rotterdam Study. Eur J Epidemiol. 2020;35(5):483–517.

6. Van Herpt TTW, Lemmers RFH, Van Hoek M, Langendonk JG, Erdtsieck RJ, Bravenboer B, et al. Introduction of the DiaGene study: Clinical characteristics, pathophysiology and determinants of vascular complications of type 2 diabetes. Diabetol Metab Syndr. 2017;9(1):1–10.

7. McKnight AJ, Patterson CC, Pettigrew KA, Savage DA, Kilner J, Murphy M, et al. A GREM1 gene variant associates with diabetic nephropathy. J Am Soc Nephrol. 2010;21(5):773–81.

8. Hadjadj S, Cariou B, Fumeron F, Gand E, Charpentier G, Roussel R, et al. Death, end-stage renal disease and renal function decline in patients with diabetic nephropathy in French cohorts of type 1 and type 2 diabetes. Diabetologia. 2016;59(1):208–16.

9. Hadjadj S, Péan F, Gallois Y, Passa P, Aubert R, Weekers L, et al. Different patterns of insulin resistance in relatives of type 1 diabetic patients with retinopathy or nephropathy: The Genesis France-Belgium study. Diabetes Care. 2004;27(11):2661–8.

10. Marre M, Jeunemaitre X, Gallois Y, Rodier M, Chatellier G, Sert C, et al. Contribution of genetic polymorphism in the renin-angiotensin system to the development of renal complications in insulin-dependent diabetes. Genetique de la Nephropathie Diabetique (GENEDIAB) study group. J Clin Invest. 1997;99(7):1585–95.

11. Mansour Aly D, Dwivedi OP, Prasad RB, Käräjämäki A, Hjort R, Thangam M, et al. Genome-wide association analyses highlight etiological differences underlying newly defined subtypes of diabetes. Nat Genet. 2021;53(11):1534–42.

12. Eastwood S V., Mathur R, Atkinson M, Brophy S, Sudlow C, Flaig R, et al. Algorithms for the capture and adjudication of prevalent and incident diabetes in UK Biobank. PLoS One. 2016;11(9).

13. Noordam R, Läll K, Smit RAJ, Laisk T, Metspalu A, Esko T, et al. Stratification of Type 2 Diabetes by Age of Diagnosis in the UK Biobank Reveals Subgroup-Specific Genetic Associations and Causal Risk Profiles. Diabetes. 2021;70(8):1816–25.

14. Levey AS, Stevens LA, Schmid CH, Zhang Y, Castro AF, Feldman HI, et al. A new equation to estimate glomerular filtration rate. Ann Intern Med. 2009;150(9):604–12.

15. Rare mutations associating with serum creatinine and chronic kidney disease. Sveinbjornsson G, Mikaelsdottir E, Palsson R, Indridason OS, Holm H, Jonasdottir A, et al. Hum Mol Genet. 2014;23(25):6935-43.
